# Fine-Grained Detection and Sorting of Fresh Tea Leaves Using an Enhanced YOLOv12 Framework

**DOI:** 10.3390/foods15030544

**Published:** 2026-02-03

**Authors:** Shuang Zhao, Chun Ye, Chentao Lian, Liye Mei, Luofa Wu, Jianneng Chen

**Affiliations:** 1 Institute of Agricultural Engineering, Jiangxi Academy of Agricultural Sciences, Nanchang 330200, China; 2 School of Computer Science, Hubei University of Technology, Wuhan 430068, China; 3 Faculty of Mechanical Engineering & Automation, Zhejiang Sci-Tech University, Hangzhou 310018, China

**Keywords:** fresh tea leaves sorting, YOLOv12, multi-scale attention, fine-grained detection, tea quality assessment

## Abstract

As the raw material for tea making, the quality of fresh tea leaves directly affects the quality of finished tea. Traditional manual sorting and machine sorting struggle to meet the requirements for high-quality tea processing. Based on machine vision and deep learning, intelligent grading technology has been applied to the automated sorting of fresh tea leaves. However, when faced with machine-picked tea leaves, the characteristics of complex morphology, small target recognition size, and dense spatial distribution can interfere with accurate category recognition, which in turn limits classification accuracy and consistency. Therefore, we propose an enhanced YOLOv12 detection framework that integrates three key modules—C3k2_EMA, A2C2f_DYT, and RFAConv—to strengthen the model’s ability to capture delicate tea bud features, thereby improving detection accuracy and robustness. Experimental results demonstrate that the proposed method achieves *precision*, *recall*, and *mAP*@0.5 of 81.2%, 90.6%, and 92.7% in premium tea recognition, effectively supporting intelligent and efficient tea harvesting and sorting operations. This study addresses the challenges of subtle fine-grained differences, small object sizes, variable morphology, and complex background interference in premium tea bud images. The proposed model not only achieves high accuracy and robustness in fine-grained tea bud detection but also provides technical feasibility for intelligent fresh tea leaves classification and production monitoring.

## 1. Introduction

Tea is a natural beverage that is rich in various nutrients, including tea polyphenols, alkaloids, amino acids, and vitamins, which can help refresh the mind, improve focus, and reduce anxiety. Additionally, regular tea consumption may offer long-term benefits such as anti-inflammatory, anti-aging, antioxidant, and immune-boosting effects [1,2]. There are significant differences in the nutrient content, tastes, and market value of various grades of tea. Famous tea is typically made from tender shoots harvested by hand in the spring, such as single buds, one bud one leaf, or one bud two leaves, which contain higher levels of tea polyphenols, amino acids, and aromatic compounds [3,4]. The famous tea made from these premium leaves not only provides superior nutritional benefits but also offers a fresh, invigorating taste, a rich aroma, and an appealing appearance. As a result, their price can be tens or even hundreds of times higher than that of the ordinary tea [5,6]. In contrast, the ordinary tea, a relatively lower-grade tea, typically has less content of L-theanine and catechin, with a poor taste due to its high content of cellulose and lignin [7]. This is primarily because the raw materials for ordinary tea are often fresh leaves harvested by machines, which typically include a significant proportion of older leaves, tea stems, and broken leaves, resulting in poor overall uniformity and tenderness of the fresh leaves [8]. The current method of harvesting famous tea is primarily manual, which is both inefficient and costly, leading to limited production [9]. Although machine harvesting significantly increases the efficiency and production of tea picking, it results in a mix of different grades of fresh tea leaves (FTLs) that cannot be directly used as raw materials for high-quality teas. Consequently, tea harvested by machine often faces challenges such as reduced quality and lower economic returns.

To enhance the economic benefits of the tea industry, the “post grading” processing model is commonly employed, in which finished tea products are classified using techniques such as winnowing and color sorting based on indicators including appearance, color uniformity, and impurity content [10,11]. However, this approach adversely affects tea flavor, morphological integrity, quality consistency, and costs. The physical characteristics of FTLs—such as tenderness, leaf thickness, moisture content, and the concentrations of polyphenols and amino acids—directly determine the appropriate processing parameters and the ultimate quality of the product [12]. When leaves of heterogeneous grade are processed together under uniform parameters, inconsistencies in product quality inevitably arise. For instance, during the wilting stage, tender leaves and mature leaves exhibit different rates of water loss [13]. Uniform processing may lead to over drying of tender leaves, causing undesirable “reddening” or loss of aroma [14]. Although the FTLs undergo similar basic processing steps, variations in fresh leaf quality and processing parameters result in substantial differences in final grade and price [15,16]. Therefore, the scientific grading of fresh leaves before processing allows for the adjustment of key parameters—such as wilting time and temperature, rolling pressure and duration, and drying temperature profiles—according to the tenderness and uniformity of different leaf categories [17]. This practice helps mitigate quality fluctuations caused by raw material inconsistency, thereby enhancing the stability, flavor harmony, and nutrient retention of the finished tea. Fresh leaf grading not only optimizes raw material utilization and standardizes processing operations, but also contributes to the control of safety-related indicators, such as the uniformity of pesticide residues [10]. From an industrial standpoint, the implementation of fresh leaf grading is essential for achieving standardized and large-scale tea production, and serves as a key technical driver in advancing the tea industry toward higher quality and greater added value.

Traditional FTL sorting methods, such as winnowing, drum screening, and vibration screening, can classify the machine-harvested FTLs and separate impurities [18,19]. However, these methods rely on the physical characteristics of raw materials, such as size, quality, density, and shape, which can lead to issues such as clogging, entanglement, damaged leaves, and inadequate accuracy, which makes it challenging for the selected FTLs to meet the purity and uniformity standards required for producing high-quality tea [20]. With the development of computer technology and machine vision, traditional methods for sorting FTLs are undergoing revolutionary changes [21,22]. Machine learning technology provides the possibility for intelligent recognition and sorting of FTLs. Song et al. developed a least-squares support vector machine model based on the shape feature histogram to identify seven grades of Keemun black tea, and its recognition accuracy reached 95.71% [23]. Lin et al. proposed a radial basis kernel support vector machine (RBF-SVM) with optimal penalty parameter selection for the automatic classification and recognition of 14 image features of Wuyiyan tea leaves and achieved an improved fresh leaf recognition rate equal to 91.00% [24]. Gan et al. used an improved genetic algorithm to select optimal 28-dimensional feature combinations for three classifiers, with SVM achieving 97% recognition accuracy [25]. Traditional machine learning algorithms, which rely on manually designed features and have limited capacity for processing massive datasets, struggle to achieve efficient and accurate automated real-time sorting of FTLs.

The integration of deep learning technology with computer vision supports the intelligent recognition and real-time sorting of FTLs [26]. Based on neural networks, deep learning algorithms can fast recognize and classify the FTLs by analyzing the geometric shapes and color textures present in fresh tea images [27]. Gao et al. utilized a convolution neural network (CNN) with a seven-layer network structure to recognize FTL images, which can effectively sort tea leaves into several types, such as single buds, single buds with single leaves, single buds with two leaves, single buds with three leaves, and single leaves and leaf stalks, and its identification accuracy was more than 90% [28]. Cao et al. compared the recognition results of four types of models (YOLOv5, YOLOv3, Fast RCNN, and SSD), and found that YOLOv5, with guaranteed recognition accuracy, had a recognition speed of 4.7 ms/frame (about four times that of the second-ranked YOLOv3). Creating the complete moisture content training set allowed the model with YOLOv5 to achieve an overall sorting accuracy of 85.4% [7]. The YOLO algorithm provides the possibility for dynamic recognition and real-time sorting of tea fresh leaf types. Based on an end-to-end architecture, it has the ability to quickly and accurately process image data, and can capture diverse features of tea fresh leaves in complex scenes with uneven lighting. Based on this, integrating deep learning with mechanical systems enables real-time recognition, classification, and automated sorting of FTLs. Chen et al. developed an automatic sorting machine using a vision-based recognition method to extract high-quality FTLs from the raw FTLs, achieving a sorting success rate of approximately 80% and an efficiency of about 15 kg/h [10]. Zhang et al. used CNN and regional segmentation for rapid machine-picked FTL identification, achieving 99.82% validation accuracy after 150 iterations, with an average sorting accuracy of 89% (max 92%) across four experiments [20]. Gan et al. developed a productive piece of equipment for sorting famous tea raw materials to meet the small-scale production needs. An air-blowing platform utilizing machine vision was designed to recognize and sort the manually picked FTLs, achieving a recognition rate, purity, selection rate, and integrity rate of 100% (98.5%), 94.32% (87.47%), 91.67% (90.67%), and 100% (100%), respectively. Under optimal parameters, the production efficiency reached 25 kg/h [29]. Although previous studies have promoted the intelligence sorting of FTLs, there remains potential for enhancement in both accuracy and speed, especially for machine-harvested FTLs. The morphological complexity, dimensional variability, diverse impurity content, small target size, and high spatial density of machine-harvested FTLs present significant challenges to algorithmic performance, particularly in terms of real-time processing, recognition accuracy, interference robustness, and multi-target classification and localization precision.

To address these challenges, we propose a novel object detection framework based on YOLOv12 tailored for machine-harvested FTLs classification. Specifically, three innovative modules are introduced: the C3k2-EMA module for enhanced multi-scale feature fusion and representation, the A2C2f_DYT module for improved perception of subtle textural and morphological details, and the RFAConv module for robust suppression of background noise and irrelevant interference. Furthermore, sophisticated data augmentation strategies are systematically integrated to bolster the model’s generalization capability across varied and unpredictable practical applications. To achieve the goal of sorting the fresh leaves of the famous tea from machine-picked tea leaves, we propose a system that combines winnowing with machine vision sorting. First, the winnowing sorting is used to separate broken leaves and fine stems. The remaining tea leaves then proceed to the machine vision sorting channel for further sorting. At the front of the sorting channel, an industrial camera is positioned to capture images of FTLs, and the imaging environment of the camera is stable. Based on this system, the enhanced YOLOv12 Framework is designed to achieve fully automated recognition and classification of FTLs into distinct quality grades (e.g., single bud, one bud one leaf, one bud two leaves, and ordinary tea leaves), aiming to significantly boost the efficiency and accuracy of premium tea sorting while minimizing dependence on manual expertise. Based on the dataset consisting of 1270 images, the enhanced YOLOv12 framework was trained and evaluated. Experimental validation demonstrates that our method outperforms existing approaches in both precision and robustness, offering an effective solution for high-quality tea classification.

## 2. Materials and Methods

### 2.1. Sample Preparation and Data Augmentation

The original images of tea were taken in the tea cultivation base of Jiangxi Academy of Agricultural Sciences (Yichun City, China) by Canon EOS 1200D (Oita Prefecture, Japan) and Sony HDR-XR350V (Shenzhen, China). The tea variety is Zhongcha 108, with a planting area of about 800 acres. To simulate the real scene of image acquisition during fresh leaf sorting, images of tea leaves on the conveyor belt were captured using a top-down photography setup with a 90° vertical shooting angle. The data collection took place within one week before and after the Qingming Festival (a traditional Chinese holiday), from 1–8 April 2025.

**Figure 1:**
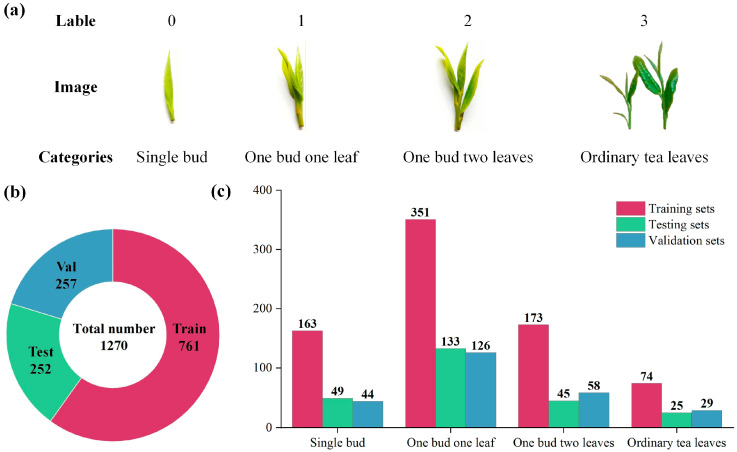
Visualization of the tea bud dataset. (**a**) Four representative categories and examples of tea leaves. (**b**) Global dataset composition of training, validation, and testing subsets. (**c**) Sample distribution of each category across the three subsets.

As shown in Figure 1, we present an overview of the tea bud dataset used in this study. Specifically, Figure 1a illustrates four representative categories, single-bud, one-bud-one-eaf, one-bud-two-leaves, and ordinary tea leaves—each labeled from 0 to 3. Figure 1b shows the overall data distribution. Figure 1c further details the sample allocation across categories for each subset. The dataset in this paper contains a total of 1270 tea images, which can be divided into four groups: single bud (256), one bud one leaf (OBOL) (510), and one bud two leaves (OBTL) (276), and ordinary tea leaves (128) which contain tatter leaves and the leaves more than two leaves with one bud. The quantity of each type of dataset is randomly distributed during a tea picking process.

To ensure the robustness of the model, the collected images were divided into training, validation, and test sets with a ratio of 3:1:1, which provides sufficient training samples while enabling reliable evaluation. The test set was kept completely independent of the training process to guarantee objectivity and generalization. In addition, multiple data augmentation strategies were employed, including basic operations such as rotation, Hue Saturation Value (HSV) color transformation, and scaling, as well as advanced techniques such as mosaic augmentation, random erasing, mixup, and copy–paste, thereby enhancing the model’s adaptability to diverse scenarios and complex backgrounds.

### 2.2. Overview of the Network

**Figure 2:**
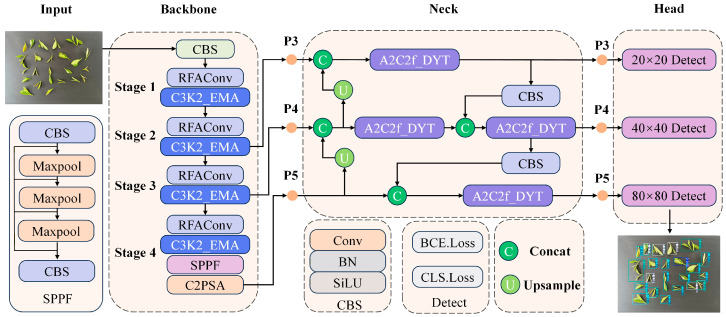
The overall architecture of the proposed tea bud detection model. The network comprises three main components: the backbone, the neck, and the head. The backbone is constructed with a series of novel modules, RFAConv, C3k2_EMA, SPPF, and C2PSA to enhance feature extraction. The neck integrates multi-level features through the A2C2f_DYT fusion modules. The head consists of three detection branches. The structure employs standard convolution, batch normalization (BN), and SiLU activation functions. The BCE Loss and Cls Loss represent the bounding-box regression and classification objectives, respectively.

To address the challenges of fine-grained classification and small-object detection in tea leaf grading, we design a novel detection framework based on the Yolov12 as a baseline, incorporating several dedicated modules to enhance feature representation, detail perception, and robustness. As shown in Figure 2. The backbone is designed to progressively extract hierarchical features from raw tea leaf images. Each stage integrates RFAConv [30] and C3K2_EMA modules to strengthen spatial and channel interactions and multi-scale attention, ensuring that fine morphological cues are effectively captured. At the end of Stage 4, a SPPF module aggregates multi-scale receptive fields, while the C2PSA module further enriches global contextual information. In the neck, to fuse multi-level features from different scales, we introduce the A2C2f_DYT module, which dynamically enhances detail perception and improves gradient propagation across scales. Together with lightweight convolutional, concatenation, and up sampling operations, the neck facilitates robust feature interaction across P3, P4, and P5 layers, thereby boosting detection of small and overlapping tea leaves. The detection head consists of parallel 20 × 20, 40 × 40, 80 × 80 detection layers applied at three scales P3, P4, and P5. This multi-scale design ensures accurate prediction of tea leaf categories with varying sizes and shapes, from single bud to multi-leaf structures. The proposed architecture enhances feature extraction with RFAConv and C3K2_EMA, improves multi-scale fusion via A2C2f_DYT, and achieves robust, high-precision performance under complex backgrounds, making it well suited for automated tea leaf grading.

#### 2.2.1. C3K2_EMA Module

**Figure 3:**
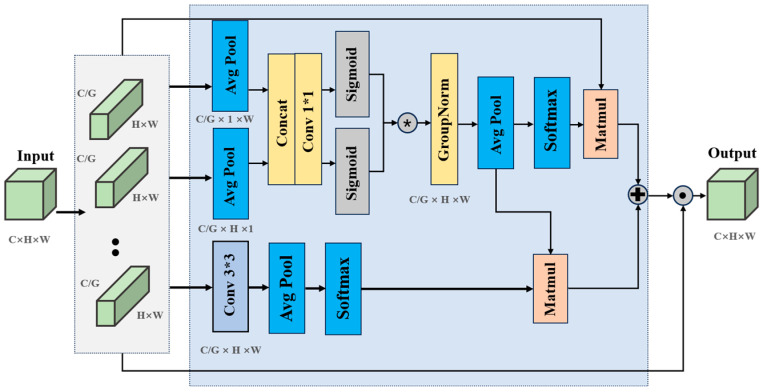
The architecture of the C3K2_EMA module. This module integrates multi-scale and directional attention to enhance fine-grained feature extraction. The sign represent multiplication.

To address subtle visual challenges in tea leaf recognition, such as minute shape variations and indistinct differences between leaves of similar grades, we introduced the C3K2_EMA module to enhance the model’s sensitivity to fine-grained leaf features and structural information. Although conventional convolutional modules can extract local features effectively, they often struggle to capture fine-grained directional cues and long-range dependencies, which are critical for distinguishing subtle morphological differences in tea leaves. To overcome these limitations, we design the C3K2_EMA module as shown in Figure 3, an efficient multi-scale attention module that integrates directional context and cross-spatial interactions into feature representation.

The C3K2_EMA module is an efficient multi-scale attention module that simultaneously incorporates directional context and cross-spatial dependencies into feature representation. As shown in Figure 3, given an input feature map $X\in R^{B\times C\times H\times W}$, the channels are evenly divided into *G* groups, containing $\frac{C}{G}$ channels, resulting in the grouped features $X_{g}\in R^{(B\cdot G)\times \frac{C}{G}\times H\times W}$. To model contextual information along row and column directions, average pooling is performed along the horizontal and vertical axes:

(1) $$X_{h}\;={\;\mathrm{Pool}}_{h}\left(X_{g}\right),\;{\;X}_{w}\;={\;\mathrm{Pool}}_{w}(X_{g})$$

After concatenation and a 1 × 1 convolution, the descriptors are split and activated by sigmoid functions to generate directional gating weights:

(2) $$G_{h}=\sigma ({Conv}_{1\times 1}\left(X_{h}\right)),\;{\;G}_{w}={\sigma (Conv}_{1\times 1}(X_{w}))$$

The gated features are obtained as:

(3) $$X_{1}=GN\left(X_{g}⊙G_{h}⊙G_{w}\right)$$

where ⊙ denotes element-wise multiplication and GN denotes Group Normalization. In the parallel branch, the grouped features are processed by a 3 × 3 convolution to retain local spatial structures:

(4) $$X_{2}\;=\;{\mathrm{Conv}}_{3\times 3}\left(X_{g}\right)$$

Global dependencies are captured by constructing two complementary attention maps using global average pooling (*GAP*) and *Softmax*:

(5) $$A\;=\;\mathrm{Softmax}(\mathrm{GAP}\;(X_{1}))●\mathrm{Reshape}\;(X_{2})$$

(6) $$B=\mathrm{Softmax}(\mathrm{GAP}\;(X_{2}))●\mathrm{Reshape}\;(X_{1})$$

The fused spatial attention map is then obtained as:

(7) $$W=\sigma (A+B)\in R^{1\times H\times W}$$

Finally, the spatial weight mask is applied to the original grouped features:

(8) $$Y_{g}=X_{g}⨀W$$

and the groups are aggregated back to the full channel dimension, yielding the output feature map $Y\in R^{B\times C\times H\times W}$. As a result, the C3K2_EMA module helps the model more reliably distinguish visually similar tea leaf categories by emphasizing discriminative structural cues. This design significantly strengthens the feature representation and boosts recognition performance in tea bud detection tasks.

#### 2.2.2. A2C2f_DYT Module

**Figure 4:**
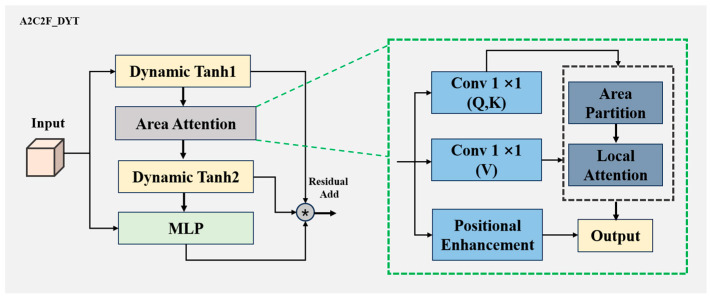
The architecture of the A2C2f_DYT module. This module integrates DynamicTanh activations into both area attention and feed forward branches, which improve multi-scale feature fusion ability. The asterisks represents “Residual Add”.

To enhance the recognition stability of tea buds and leaves under stacked and multi-scale conditions during tea leaf inspection, we introduced the A2C2f_DYT module to strengthen adaptive feature fusion. In tea leaf detection tasks, existing attention fusion modules can integrate multi-scale features but often suffer from insufficient nonlinearity, particularly when dealing with fine-grained small-object recognition. To address this issue, we introduce the A2C2f_DYT module as shown in Figure 4, which integrates DynamicTanh (*DyT*) activations [31] into both the attention and feed-forward branches, enabling dynamic nonlinearity regulation. The main formula is as follows:

$$DyT\left(x\right)=\omega \times Tanh\left(𝜕x\right)+b$$

where 𝜕 is a learnable global scaling parameter which controls the smoothness or steepness of the Tanh curve. ω and b are per-channel weights and biases, used to adjust the response magnitude of each channel.

In Figure 4, we give an input feature map $X\in R^{\left(B,C,H,W\right)}$, and the *DyT* activation is first applied before area-attention:

$$X^{\prime }=X+Attn(DyT_{1}(X))$$

where *Attn(.)* denotes the area-attention operator and *DyT*1 adaptively regulates the input. The output is then processed by the MLP branch with another *DyT* activation:

$$Y=X^{\prime }+MLP(DyT_{2}(X^{\prime }))$$

where residual connections ensure stable gradient propagation and prevent over-smoothing. In the complete A2C2f_DYT module, multiple ABlock-DYT units are sequentially stacked:

$$F\left(X\right)=\begin{array}{c} n\\ ⊗\;\\ i=1 \end{array}ABlock-DYT_{i}(X)$$

with the hidden dimension constrained to be a multiple of 32 for balanced multi-head computation. By jointly modeling area-attention, dynamic activation, and residual learning, the A2C2f_DYT module enhances robustness in identifying visually similar tea categories, demonstrating particularly strong performance under conditions of size variation and overlapping buds and leaves.

#### 2.2.3. RFAConv Module

**Figure 5:**
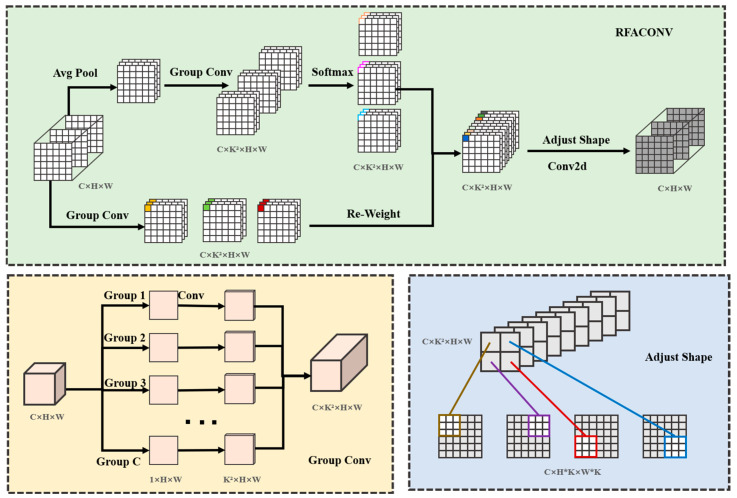
The architecture of the RFAConv module. This module emphasizes distinct features within the receptive field slider and prioritizes spatial features within the receptive field. The sign represent multiplication.

To address the challenge of detecting small and densely distributed tea buds, where fine structural details are easily lost during downsampling, we introduce the RFAConv module into the backbone network. Traditional convolutional neural networks often lose edge and detail information during down sampling. The distinction between different grades of tea buds is primarily manifested in morphological structure and texture details, such as the subtle yet critical differences between “one bud one leaf” and “one bud two leaves.” To address these issues, this study incorporates the RFAConv module into the backbone network of the YOLOv12 framework, as shown in Figure 5. The RFAConv module emphasizes distinct features within the receptive field slider and prioritizes spatial features within the receptive field. This approach resolves the problem of convolutional kernel parameter sharing.

In RFAConv, we use Group Conv to extract spatial features. This method is faster and more efficient than the original Unfold method. Specifically, the input image $X\in R^{B\times C\times H\times W}$ is split into two branches. In the first branch, we use AvgPool2d to aggregate the global information of each receptive field feature to reduce computational overhead and parameter count. Then, we apply a 1 × 1 group convolution operation to obtain an attention map $A_{rf}=R^{B\times 9C\times H\times W}$. Finally, we apply a softmax function to emphasize the importance of each feature within the receptive field. The computation of the attention map is represented as follows:

$$A_{rf}=Softmax(G^{1\times 1}(AvgPool2d(X)))$$

where $A_{rf}$ denotes the attention map, $G^{1\times 1}$ denotes a 1 × 1 batch convolutional operation, and *X* denotes the input feature map.

In the second branch, we apply Group Conv directly to the input image $X=R^{B\times C\times H\times W}$. After applying normalization and activation functions, we obtain the receptive field feature map $F_{rf}=R^{B\times 9C\times H\times W}$, which is calculated as follows:

$$F_{rf}=RELU(Norm(G^{k\times k}\left(X\right))$$

where $F_{rf}$ denotes the receptive field feature map, $G^{k\times k}$ represents batch convolutions with kernel size k, Norm indicates normalization, and X denotes the input feature map.

Finally, the feature maps are reweighted using the obtained attention map. Then, the feature shape and convolution operation are adjusted to obtain the final input result. The computational representation is as follows:

The performance of convolutional neural networks is constrained by standard convolution operations, compared to traditional modules. Convolutions rely on shared parameters and are insensitive to subtle differences in detailed information, such as the difference between “one bud one leaf” and “one bud two leaves”. However, RFAConv improves the ability to recognize fine details by emphasizing different features within the receptive field slider and prioritizing spatial features to improve the detection of small objects. Additionally, the weighted aggregation mechanism of the attention map suppresses noise by utilizing feature information from each receptive field to compensate for the shortcomings of existing attention mechanisms in complex backgrounds. The RFAConv module improves the detection of small and overlapping tea buds by preserving fine-grained spatial details while suppressing background noise.

$$Output=Conv(rearrange(F_{rf}\times A_{rf}))$$

## 3. Model Training and Experimentation

### 3.1. Network Training

All experiments were conducted on a Windows-based workstation equipped with an NVIDIA GeForce RTX 4090 graphics processing unit with 24 gigabytes of memory. The implementation environment was based on Python version 3.12.7 and PyTorch version 2.6.0. The entire training process was carried out within the PyTorch deep learning framework. During training, all input images were resized and standardized to a fixed resolution of 640 by 640 pixels. The batch size was consistently set to 32, and the model was trained for up to 300 epochs. An early stopping mechanism was employed to halt training if the validation performance did not improve for 100 consecutive epochs. The AdamW optimization algorithm was used, with an initial learning rate of 0.00125 and a momentum parameter of 0.9. The model weights corresponding to the highest validation accuracy were saved as the final output for subsequent evaluation.

### 3.2. Evaluation Criteria

To comprehensively evaluate the performance of the proposed model on tea detection, several standard evaluation metrics were adopted, including precision, recall, F1-score, mean average precision (*mAP*), parameter count (*Params*), computational complexity (*GFLOPs*), and inference speed measured by frames per second (FPS). The precision metric, defined in Equation (9), measures the proportion of correctly predicted positive instances among all predicted positives, and is calculated as the ratio of true positives (*TP*) to the sum of true positives and false positives (*FP*):

(9) $$Precision=\frac{TP}{TP+FP}$$

The *Recall*, shown in Equation (10), quantifies the ability of the model to identify all relevant instances, computed as the ratio of true positives to the sum of true positives and false negatives (*FN*):

(10) $$Recall=\frac{TP}{TP+FN}$$

The *F*1-*score*, presented in Equation (11), is the harmonic mean of precision and recall, providing a balanced measure of the *precision* and *recall*:

(11) $$F1score=\frac{2\times Precision\times Recall}{Precision+Recall}$$

To assess overall detection performance, the mean average precision (*mAP*) at an intersection-over-union threshold of 0.5 is utilized, which is a standard evaluation metric widely used in object detection tasks. As shown in Equation (12), *mAP* is calculated by integrating the precision–recall curve for each class and averaging over all classes:

(12) $$mAP=\frac{\sum_{n}\int_{0}^{1}p(r)dr}{n}$$

Additionally, the model’s efficiency is evaluated using the number of parameters (*Params*) and *GFLOPs*, which reflect the model’s complexity and computational cost, respectively. The *FPS* metric is employed to measure the inference speed and real-time capability of the model.

## 4. Results and Discussion

### 4.1. Performance

**Figure 6:**
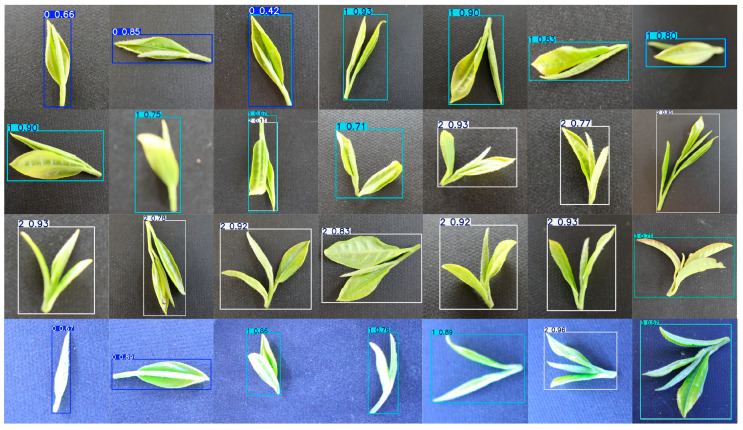
Visualization of the detection results for the tea bud dataset. The figure shows the bounding boxes generated by the proposed model, where each color box represents a detected tea bud category with the corresponding confidence score. The results demonstrate that the model can accurately localize and classify different types of tea buds and leaves under varying lighting and background conditions, maintaining high robustness.

To comprehensively evaluate the effectiveness and detection capability of the proposed model, we visualize the detection results obtained from the proposed tea bud detection model. In Figure 6, representative samples from the testing set show each color box corresponding to a detected tea bud category with its associated confidence score. It can be observed that the model accurately identifies and localizes different types of tea buds and leaves—including single bud, one bud one leaf, one bud two leaves, and ordinary tea leaves—under various illumination and background conditions. Even in complex scenarios involving overlapping leaves or partial occlusion, the proposed model maintains stable detection confidence and precise bounding box localization. These results demonstrate the strong robustness and generalization ability of the model in handling diverse field environments, providing reliable support for automated tea picking and intelligent tea quality assessment.

### 4.2. Quantitative Analysis

To further validate the performance of the proposed model, we conducted a series of quantitative analyses to comprehensively evaluate its accuracy, robustness, and generalization capability across multiple detection tasks. As presented in Table 1, the proposed model demonstrates strong detection performance across all four categories of FTLs, with an average *mAP* of 92.7% on the test set and 90.4% on the validation set. This indicates that the proposed model achieves high detection accuracy and exhibits excellent capability in both tea bud recognition and localization. Among the four classes, “one bud one leaf” achieved the highest detection accuracy, reaching a *mAP* of 96.0%, a *precision* of 85.7%, and a *recall* of 98.0% on the test set. This finding indicates that the proposed model can accurately recognize premium tea varieties, showcasing its remarkable ability to identify and localize high-quality tea leaves with fine-grained visual distinctions. Notably, the performance on the test set remained comparable or even slightly improved compared to the validation set, particularly for the “one bud with multiple leaves” category, where *precision* increased from 82.4% to 91.2%. This consistency suggests that the model is not overfitting and maintains good detection stability under unseen data. In addition, the high recall scores observed across all classes—especially the 98.0% *recall* for “one bud one leaf” and 93.4% for “single bud”—highlight the model’s ability to minimize missed detections, which is critical for reliable field application. Encouragingly, our model exhibited high *mAP* accuracy when distinguishing premium tea categories such as single bud, one bud one leaf, and one bud two leaves. This demonstrates that the proposed model can accurately recognize and localize high-grade FTLs, aligning well with our research objective of promoting intelligent harvesting efficiency for premium tea production. However, the “one bud two leaves” class showed relatively lower *precision* (70.7% on the test set), although its *recall* (91.6%) and *mAP* (92.3%) remained satisfactory. This discrepancy is likely due to the visual similarity between this class and “one bud with multiple leaves,” as well as its limited representation in the training data. Future work will aim to address these limitations, particularly for underrepresented classes and exploring class-specific augmentation strategies. Enhancing the model with more discriminative feature extraction modules may also contribute to more accurate fine-grained classification.

To evaluate the efficiency and detection capability of the proposed tea bud recognition algorithm, a series of comparative experiments was conducted against several mainstream object detection frameworks, including YOLOv6, YOLOv8, YOLOv9, YOLOv10, YOLOv11, and YOLOv12. All models were trained and tested under identical hyperparameter settings and hardware environments to ensure a fair comparison. As presented in Table 2, the proposed method achieves the highest *mAP* of 92.7%, surpassing all baseline models. It also maintains a competitive *recall* of 90.6%, demonstrating superior capability in detecting small and partially occluded tea buds. Despite having comparable computational complexity with 7.7 GFLOPs and parameter count 2.92 M to YOLOv9 and YOLOv11, the proposed method achieves a notable accuracy improvement, confirming its efficiency in balancing detection precision and computational cost.

To assess the effectiveness of each proposed component, a series of ablation experiments was conducted using the YOLOv12n architecture as the baseline. As shown in Table 3. When only the C3k2-EMA module was added, which enhances multi-scale feature aggregation through efficient cross-spatial attention, the model achieved a noticeable improvement. Specifically, the *mAP* increased from 89.1 percent to 91.2 percent, and the *recall* improved to 86.0 percent. This demonstrates the module’s ability to strengthen the representation of tea bud–leaf structures across different scales. Subsequently, the A2C2f-DYT module was introduced. This module integrates a dynamic Y-shaped Transformer and a learnable activation mechanism, which together improve the model’s capability to capture fine-grained contextual dependencies. As a result, the performance further improved, reaching 92.5 percent *mAP*, with *precision* rising to 89.6 percent and *recall* to 87.4 percent. Finally, the RFAConv module was incorporated, which aligns receptive fields and applies attention-based reweighting to enhance feature representation in spatially diverse regions. With all three modules combined, the model achieved the best overall performance. The *mAP* peaked at 92.7 percent, while the *recall* reached 90.6 percent. Although the *precision* decreased slightly to 81.2%, this reflects a deliberate trade-off in favor of higher recall—a choice motivated by practical considerations in tea grading. Missing a high-value leaf results in irreversible economic loss, as it is downgraded and processed as bulk tea—potentially forfeiting over 90% of its market value. During subsequent processing stages, due to the influence of processing parameters and production processes, ordinary tea leaves develop distinct visual characteristics (e.g., curled edges, yellowing, or uneven texture) that make them easily distinguishable from genuine famous tea. As a result, these misclassified samples can be efficiently removed during routine manual inspection at minimal labor cost. The *GFLOPs* rose from 7.1 to 7.7, and the parameter count remained close to 2.92 million. The inference speed was maintained at 333 frames per second, confirming that the proposed enhancements offer a favorable balance between accuracy and efficiency. These results validate that each individual module contributes positively to detection performance, and their combination leads to synergistic improvements without compromising real-time deployment.

### 4.3. Qualitative Analysis

**Figure 7:**
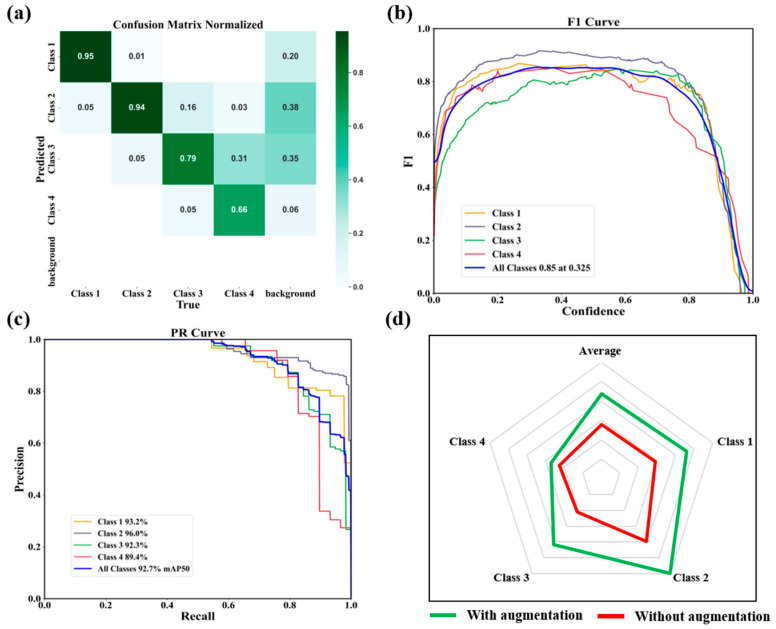
Comprehensive performance evaluation of the proposed tea bud detection model on the testing set. (**a**) The normalized confusion matrix shows the classification accuracy and cross-category misclassification rates among four tea categories. (**b**) The F1 score curve illustrates the balance between precision and recall across different confidence thresholds for each class. (**c**) The precision–recall (PR) curve further demonstrates the model’s robustness and discriminative capability, with all classes achieving an average *mAP* of 92.7%. (**d**) The radar chart compares the model’s detection performance with and without data augmentation, highlighting consistent improvement in all categories after augmentation.

To validate and evaluate the proposed tea bud detection model, we generate a confusion matrix, F1 score curves, precision–recall (PR) curves. As shown in Figure 7a, the normalized confusion matrix presents the classification results of four tea categories. The model achieves high recognition accuracy for single bud, one bud one leaf, and one bud two leaves, with prediction accuracies of 95%, 94%, and 79%, respectively. Slight confusion occurs between one bud two leaves and ordinary tea leaves, primarily due to their similar morphological characteristics and overlapping structures in natural conditions. Nevertheless, the overall classification results confirm that the proposed model maintains high stability and discriminative capability across all classes. As shown in Figure 7b. The F1 score curves illustrate the relationship between confidence and detection accuracy. The curves corresponding to single bud and one bud one leaf remain consistently above 0.85 across most confidence intervals, indicating strong and stable detection capability. This demonstrates that the model effectively balances precision and recall, maintaining robust performance even under varying confidence thresholds. As shown in Figure 7c, the PR curves further highlight the discriminative power of the model. The PR curve of the one-bud-one-leaf class shows the largest enclosed area, followed by buds, indicating superior precision and recall. The overall *mAP* reaches 92.7%, confirming that the proposed model achieves high detection accuracy and low false detection rates in complex lighting and background environments. These findings collectively demonstrate that the proposed method can accurately detect and classify fine-grained morphological variations in tea buds, laying a solid foundation for intelligent tea harvesting and quality evaluation systems. Finally, the radar chart in Figure 7d compares the model’s detection performance with and without data augmentation. When only basic transformations such as HSV adjustment, rotation, and translation are applied, the model exhibits relatively limited accuracy and generalization. However, after introducing advanced augmentation strategies, including mosaic, mix-up, and copy–paste, the detection performance improves significantly across all categories, particularly for the ordinary tea leaves class, which previously showed weaker performance. The average *mAP* increases by 3.1%, and the *mAP* of each category improves by 3.4%, 4.1%, 4.1%, and 0.9%, respectively, demonstrating the positive impact of multi-type data augmentation on model robustness.

## 5. Conclusions

With the growing demand for high-quality tea products and the increasing emphasis on automation in agricultural production, intelligent FTL classification has become a critical research topic in precision tea processing and digital management. This study proposes an improved FTLs detection framework based on YOLOv12 that integrates RFAConv, C3K2_EMA, and A2C2f_DYT modules to enhance the model’s ability in multi-scale feature extraction and contextual interaction. The RFAConv module introduces re-parameterized feature aggregation to strengthen spatial representation, while the C3K2_EMA module incorporates directional and cross-spatial attention to refine feature responses across different orientations. Furthermore, the A2C2f_DYT module adaptively fuses multi-level features, improving global-local semantic consistency within the network. These innovations collectively enable the model to accurately identify tea buds and distinguish subtle morphological differences among similar categories under complex background conditions. Extensive experiments on the self-built tea bud dataset demonstrate the superiority of the proposed method. Experimental results demonstrate that the proposed method achieves precision, recall, and mAP@0.5 of 81.2%, 90.6%, and 92.7% in premium tea recognition, with significant improvements in precision and recall across all categories compared with the baseline. Ablation experiments further verify that each introduced module contributes positively to detection performance, particularly in enhancing feature representation for small or overlapping tea buds. The proposed model not only achieves high accuracy and robustness in fine-grained tea bud detection but also provides technical feasibility for intelligent FTLs classification and production monitoring.

While our method demonstrates strong performance under controlled conditions, several practical limitations should be acknowledged when considering real-world deployment. The model requires a GPU with at least 16 GB of memory to achieve real-time inference at full resolution, which may be cost-prohibitive for small-scale or legacy sorting systems. In addition, camera lenses must be cleaned regularly to prevent dust accumulation, and lighting conditions may require periodic calibration. Future research will focus on integrating lightweight transformer architectures and multi-view imaging systems to build real-time detection models adaptable to the automated sorting platform. This work contributes to the development of smart agricultural technologies and lays a foundation for the digital transformation of the tea industry.

## Figures and Tables

**Table 1:** Class-wise detection *precision*, *recall*, and *mAP* of the proposed model.

Classes	*Precision* (%)		*Recall* (%)		*mAP* (%)	
	Validation	Test	Validation	Test	Validation	Test
Class 1	87.3	77.4	89.8	93.4	93.9	93.2
Class 2	**90.5**	85.7	**94.0**	**98.0**	**96.8**	**96.0**
Class 3	74.1	70.7	88.9	91.6	88.1	92.3
Class 4	82.4	**91.2**	75.0	79.3	82.9	89.4
Average	83.6	81.2	86.9	90.6	90.4	92.7

Bold represents optimal.

**Table 2:** The quantitative comparison with mainstream object detection methods on the tea dataset.

Methods	*mAP* (%)	*Precision* (%)	*Recall* (%)	*GFLOPs*	*Params* (*M*)	*FPS*
YOLOv6 [32]	89.4	82.1	87.3	11.8	4.23	353
YOLOv8 [33]	89.0	82.7	81.1	8.1	3.00	364
YOLOv9 [34]	92.6	82.1	87.3	7.6	**1.97**	298
YOLOv10 [35]	87.6	73.9	86.4	8.2	2.69	**556**
YOLOv11 [36]	90.0	**83.3**	84.3	7.6	2.90	369
YOLOv12 [37]	89.1	83.0	80.5	**7.1**	2.83	261
Proposed Method	**92.7**	81.0	**90.6**	7.7	2.92	294

Bold represents optimal.

**Table 3:** Comparison of ablation experiments within the proposed model.

Baseline	EMA	DYT	RFA	*mAP* (%)	*Precision* (%)	*Recall* (%)	*GFLOPs*	*Params* (M)	*FPS*
YOLOv12n				89.1	83.0	80.5	**7.1**	**2.83**	**333**
	√			91.2	84.6	86.0	7.6	2.91	322
	√	√		92.5	**89.6**	87.4	7.4	2.87	312
	√	√	√	**92.7**	81.2	**90.6**	7.7	2.92	**333**

Bold represents optimal. The “√” represents the model include this module.

## Data Availability

The data that support the findings of this study are available upon request from the corresponding author. The data are not publicly available due to privacy or ethical restrictions.
